# Effect of exercise-induced muscle damage on muscle hardness evaluated by ultrasound real-time tissue elastography

**DOI:** 10.1186/s40064-015-1094-4

**Published:** 2015-07-02

**Authors:** Osamu Yanagisawa, Jun Sakuma, Yasuo Kawakami, Katsuhiko Suzuki, Toru Fukubayashi

**Affiliations:** Faculty of Business and Information Sciences, Jobu University, 634-1 Toyazuka-machi, Isesaki, Gunma 372-8588 Japan; Department of Health Science, Musashigaoka Junior College, 111-1 Minamiyoshimi, Yoshimi-cho, Hiki, Saitama 355-0154 Japan; Faculty of Sport Sciences, Waseda University, 2-579-15 Mikajima, Tokorozawa, Saitama 359-1192 Japan

**Keywords:** Real-time tissue elastography, Magnetic resonance imaging, Muscle hardness, Eccentric contraction, Muscle damage, Muscle edema

## Abstract

**Purpose:**

To assess the effect of exercise-induced muscle damage on muscle hardness and evaluate the relationship between muscle hardness and muscle damage indicators.

**Methods:**

Seven men (mean 25.3 years; 172.7 cm; 66.8 kg) performed the single-leg ankle plantar flexion exercise involving both concentric and eccentric contractions (10 sets of 40 repetitions). The hardness of the medial gastrocnemius (MG) was evaluated using ultrasound real-time tissue elastography before, from day 1 to 4, and day 7 after exercise. The strain ratio between the MG and a reference material was calculated. Simultaneously, we evaluated the magnetic resonance T2 value (an index of edema) of the triceps surae, the ankle dorsiflexion range of motion (ROM), and calf muscle soreness. Serum creatine kinase activity was assessed before, 2 and 4 h, and from day 1 to 4 after exercise.

**Results:**

The MG showed lower strain ratio, indicating increased muscle hardness, on day 4 post-exercise (P < 0.01) and higher T2 values on days 1–7 post-exercise (P < 0.01) relative to each pre-exercise value. The ankle dorsiflexion ROM was lower on days 2–4 post-exercise (P < 0.01). The serum creatine kinase markedly increased on days 3 and 4 post-exercise (not significant). The degree of muscle soreness among the post-exercise time points was similar. The decreased strain ratio did not correlate with the increased T2, the decreased joint ROM or muscle soreness.

**Conclusion:**

Muscle hardness increased after strenuous resistance exercise, but the change was not related with muscle edema, decreased joint ROM, or muscle soreness resulting from muscle damage.

## Background

Eccentric muscle contraction, in which muscle fibers stretch during force generation, creates high tension in muscle fibers and is more likely to result in contraction-induced muscle damage (Friden et al. 1983; Jones et al. 1986; Beaton et al. 2002). Several studies have reported increased muscle stiffness along the longitudinal axis of the muscle (increased muscle tension) as a pathophysiological response to eccentric exercise (Howell et al. 1993; Murayama et al. 2000; Whitehead et al. 2001; Hoang et al. 2007; Reisman et al. 2009; Yanagisawa et al. 2011a). This increased longitudinal muscle stiffness may be related to decreased joint range of motion (ROM) (Nosaka and Clarkson 1996; Murayama et al. 2000; Nosaka et al. 2001; Ingham et al. 2010). In addition, a few studies have observed elevated muscle hardness (an elastic property that represents transverse muscle stiffness) after eccentric exercise, using a tissue pressure method that applies pressure perpendicular to the skin surface (Murayama et al. 2000; Andersen et al. 2006). Murayama et al. (2000) observed elevated muscle hardness and decreased joint ROM after eccentric exercise and suggested that the elevated muscle hardness stems in part from increased longitudinal muscle stiffness resulting from muscle damage. However, the hardness within a specific muscle before and after repetitive eccentric muscle contraction and the relationship between post-exercise muscle hardness and muscle damage indicators have not been fully elucidated.

In contrast to the tissue pressure method, ultrasound real-time tissue elastography (RTE) can assess the hardness distribution within a specific muscle (Drakonaki and Allen 2010; Niitsu et al. 2011; Yanagisawa et al. 2011b; Chino et al. 2012). In living humans, light epidermis compression with a hand-held ultrasound transducer produces strain within subcutaneous tissue. The strain rate is lower in harder tissue than in softer tissue (Drakonaki and Allen 2010; Niitsu et al. 2011; Yanagisawa et al. 2011b; Chino et al. 2012). RTE visualizes the distribution of the strain within the tissue as a translucent color-coded tissue elasticity map superimposed on the B-mode image. We can semi-quantitatively assess the hardness of a specific tissue by comparing its strain rate with that of a reference material (strain ratio) (Niitsu et al. 2011; Yanagisawa et al. 2011b; Chino et al. 2012). Using RTE, Niitsu et al. (2011) reported that the hardness of the biceps brachii muscle was significantly higher for several days after eccentric elbow flexion exercise. However, the study of Niitsu et al. (2011) did not clarify the relationship between muscle hardness and muscle damage indicators.

Magnetic resonance (MR) T2-weighted image and its coefficient, the T2 value, have frequently been utilized for evaluation of muscle damage resulting from repeated eccentric muscle contraction (Nosaka and Clarkson 1996; Nosaka et al. 2001; Yanagisawa et al. 2003b, 2011a; Guilhem et al. 2013; Lacourpaille et al. 2014; Schuermans et al. 2014; Fulford et al. 2015). Repetitive eccentric muscle contraction often results in damage-related muscle edema (Friden et al. 1988). Fluid accumulation prolongs the T2 value of an exercised muscle and increases the signal intensity on T2-weighted image (Yanagisawa et al. 2003a). Intramuscular fluid accumulation would be expected to elevate intramuscular pressure (Friden et al. 1986, 1988; Crenshaw et al. 2000). Friden et al. (1986) suggested that the greater intramuscular pressure observed 2 days after eccentric exercise was attributable in part to the decreased compliance of the compartment resulting from damage-related muscle edema. In addition, a positive correlation was observed between intramuscular pressure and hardness (Steinberg 2005; Steinberg et al. 2011). Therefore, although the damage-related muscle edema is assumed to contribute to the elevated muscle hardness after eccentric exercise, the exact relationship remains unclear.

We aimed to investigate the effect of exercise-induced muscle damage on muscle hardness using RTE. A secondary aim was to determine the associations between muscle hardness and muscle damage indicators (decreased joint ROM, edema, and soreness) after strenuous resistance exercise. We postulated that strenuous resistance exercise increases muscle hardness and that there are pathophysiological relationships between increased muscle hardness and muscle damage indicators.

## Methods

### Subjects

The participants in this study were seven healthy men (mean ± standard deviation (SD): age, 25.3 ± 1.5 years; height, 172.7 ± 5.9 cm; weight, 66.8 ± 7.2 kg) who had not performed lower extremity resistance training in the previous 6 months. The right leg was dominant in all subjects (defined as the preferred kicking leg). Non-resistance trained subjects were chosen to exclude the possibility of protective effects against muscle damage (Nosaka et al. 2001). In addition, none of the subjects had a history of muscle strain in the lower extremity. All subjects were instructed to refrain from participating in any physical exercise beginning 1 week prior to the measurements and from undergoing private physical therapeutic activity or taking any supplements during the measurements.

### Exercise protocol

**Figure 1:**
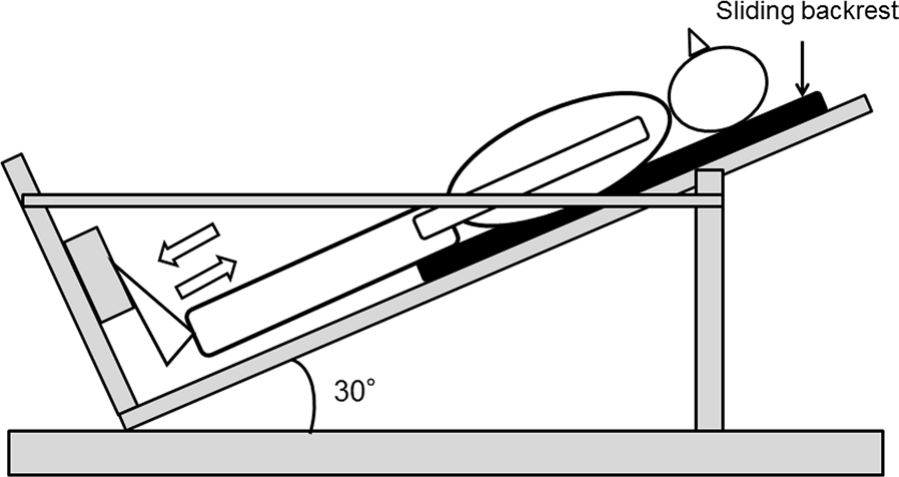
Schematic diagram of the dynamic ankle plantar flexion exercise. The backrest smoothly slid in accordance with the subject’s ankle movement.

Each subject rested on an exercise device specially designed for ankle plantar flexion, with the knee joint extended and the metatarsal bone resting on a stool (Figure 1) (Kanda et al. 2013). The slope of the backrest was 30°, so that the exercise load corresponded to approximately half of the subject’s weight (exercise load = body mass × sin 30°). With their right leg, subjects performed single-leg ankle plantar flexion exercise consisting of 10 sets of 40 repetitions with a 3-min rest between sets. The ROM of the ankle joint during the exercise was maintained between 20° (dorsiflexion position) and 15° (plantar flexion position) using an electronic goniometer (SG110/A, Biometrics, Newport, UK) with its ends attached to the distal-lateral part of the fibula and the lateral part of the foot. Each subject received visual feedback on his ankle joint ROM during the exercise via display of the joint ROM value on a personal computer. The exercise was performed in accordance with the rhythm of an electrical metronome at a speed of 60 counts/min; ankle dorsiflexion and plantar flexion were alternated and repeated every 1 s. Out of seven subjects, two could not maintain the prescribed ROM of the ankle joint in later sets, which could consequently disturb the rhythm of the exercise itself. Therefore, for these two subjects, the number of repetitions per set was reduced in order to maintain the ankle joint ROM during the exercise, and the number of sets was increased accordingly. All subjects completed a total of 400 repetitions of ankle plantar flexion.

### RTE

Axial B-mode and RTE images of the right medial gastrocnemius (MG) were obtained before and 1, 2, 3, 4, and 7 days after exercise using a digital ultrasound system (EUB-7500; Hitachi Aloka Medical, Ltd., Tokyo, Japan) with a 14–6 MHz linear array transducer (EUP-L65). In consideration of the possibility that some subjects would be unable to stretch their calf muscles fully during the post-exercise RTE scans because of severe post-exercise muscle soreness, the RTE measurements were performed within the MG in a somewhat relaxed state with the knee 45° flexed and the ankle 20° plantar flexed. We adjusted the location of the ultrasound probe on a line drawn on the muscle belly of the MG (70% of the distance between the medial point of the knee joint space and the central point of the medial malleolus) so that we could scan the center region of the MG. The location of the probe was marked with semipermanent ink on the skin surface; when the mark was fading we redrew it. The probe was consistently placed on the mark so that the RTE measurement was performed at the same position for every measurement. The scanning head of the probe was coated with transmission gel to obtain acoustic coupling.

The RTE image was obtained by manual application of light repetitive compression (rhythmical compression-relaxation cycle) with the transducer in the scan position. The compression velocity during the scan was displayed as a numerical value ranging from 1 to 6 on the edge of the RTE image. The manufacturer recommends a velocity between 3 and 5 for calculation of local strain within the field of view of the RTE scan. On the other hand, this ultrasound device lacked the ability to monitor the compressive force during the scan; therefore, a highly trained examiner performed the RTE measurement to ensure that the compressive force was as constant as possible among the scans. The reference material sample (60 × 50 × 8 mm) was placed between the transducer and the MG; this material contained a homogenous internal texture (hydrogel), and had even compressibility and conductivity for cyclic pressure to the deep muscular structures (Niitsu et al. 2011; Yanagisawa et al. 2011b).

After scanning, three RTE images from each measurement session were selected at random. The criteria for image selection included (1) a compression velocity from 3 to 5 at the moment of imaging and (2) clear color-coding of the entire RTE image. In each of the three images, rectangular regions of interest (ROI) were defined individually in the MG and the reference material. The strain rate within each ROI was measured automatically using built-in software, and a strain ratio (muscle/reference ratio: the strain rate of the MG divided by that of the reference material) was calculated for each of the three images. The strain rate in response to the compression force is physically smaller in harder tissue than in softer tissue. Therefore, as the muscle becomes harder, the strain ratio decreases. The mean value of the three trials was accepted as the strain ratio value of each measurement session. Excellent intra-observer reproducibility was confirmed in the muscle/reference ratio of the three repeated RTE measurements (Yanagisawa et al. 2011b).

### MR imaging

A 1.5-Tesla MR system (Signa EXCITE XI, GE Healthcare, Milwaukee, WI, USA) with a quadknee coil was used to obtain axial MR images of the right calf before and 1, 2, 3, 4, and 7 days after exercise. The coil was always placed in the same position on the scan table to ensure reproducibility of the MR images. The scan position was approximately equal to that on the RTE scan. Auto shimming was performed before every scan. The imaging sequence (spin-echo-type) for calculating the muscle T2 value was as follows: a single slice; repetition time, 2,500 ms; echo time, 20, 40, 60, and 80 ms; 128 × 128 matrix; number of excitations, 1; field of view, 220 mm; slice thickness, 10 mm; and acquisition time, 5 min 50 s.

ROIs were drawn around the MG, the lateral gastrocnemius (LG) and soleus (SOL) on an image with a 20-ms echo time and were then copied onto the 40, 60, and 80-ms echo time images. The signal intensity for each echo time, for each muscle, was determined. Then, T2 value was calculated via the following equation: S_n_ = S_0_ exp(−TE/T2), where S_n_ represents the signal intensity at each echo time, S_0_ is signal intensity at 0 ms, and TE is echo time.

### Ankle active ROM

Right ankle dorsiflexion ROM was measured before and 1, 2, 3, 4, and 7 days after exercise using an electronic goniometer (SG110/A, Biometrics, Newport, UK) with its ends attached to the distal-lateral part of the fibula and the lateral part of the foot. The subject was asked to dorsiflex the ankle joint voluntarily from the anatomic position (defined as 0°) with a straight knee three times, and the mean value was adopted as the value of the ankle dorsiflexion ROM. The goniometer-derived signals were transmitted to a personal computer via an A/D converter (PowerLab 16SP, ADInstruments, NSW, Australia) and analyzed using a Labchart 7 software (ADInstruments, NSW, Australia).

### Serum creatine kinase (CK) activity

Approximately 12 mL of blood was collected by venipuncture from an antecubital vein before exercise; 2 and 4 h after exercise; and 1, 2, 3, and 4 days post-exercise. The blood samples were collected into serum separation tubes. The blood in the serum separation tubes was left at room temperature for 30 min to allow clotting and centrifuged at 1,000×*g* for 10 min. The serum was then removed and immediately cooled to −80°C and stored for later analysis. Bio Medical Laboratories (BML, INC., Tokyo, Japan) measured serum CK activity spectrophotometrically using an automated analyzer (Hitachi model 7170; Tokyo, Japan) by an UV method (Nittobo Medical Co., LTD; Tokyo, Japan). According to BML’s instruction, the normal reference range for males using this method is 50–230 U/L (37°C).

### Muscle soreness

Right calf muscle soreness was assessed before and 1, 2, 3, 4, and 7 days after exercise using a 100-mm visual analog scale, on which 0 indicated no pain and 100 indicated “pain preventing one from walking unassisted”. Subjects were instructed to mark “their soreness during walking” on the visual analog scale because muscle soreness frequently occurs during mechanical stimulation of the muscle (Hoang et al. 2007; Yanagisawa et al. 2011a).

### Statistical analysis

Means and SD were calculated for all variables. Because of the small sample size and non-normal distribution of the data in this study, a non-parametric Friedman test was used to evaluate the significance of differences over time for each variable. Then, if a significant overall difference was found, a Wilcoxon test with Bonferroni correction as a post hoc test was performed to evaluate the significance of changes from the pre-exercise value for the strain ratio, T2 value, ankle dorsiflexion ROM, and serum CK activity. The degree of muscle soreness was compared among the post-exercise time points using the abovementioned post hoc test because all of the subjects reported the pre-exercise value to be 0 on the visual analog scale. Moreover, Spearman’s rank correlation coefficient (rs) was calculated to assess the correlation between the strain ratio and some indicators of muscle damage (T2 value, ankle dorsiflexion ROM, and severity of muscle soreness). The level of statistical significance was set at P < 0.05 for all analyses.

## Results

### RTE

**Figure 2:**
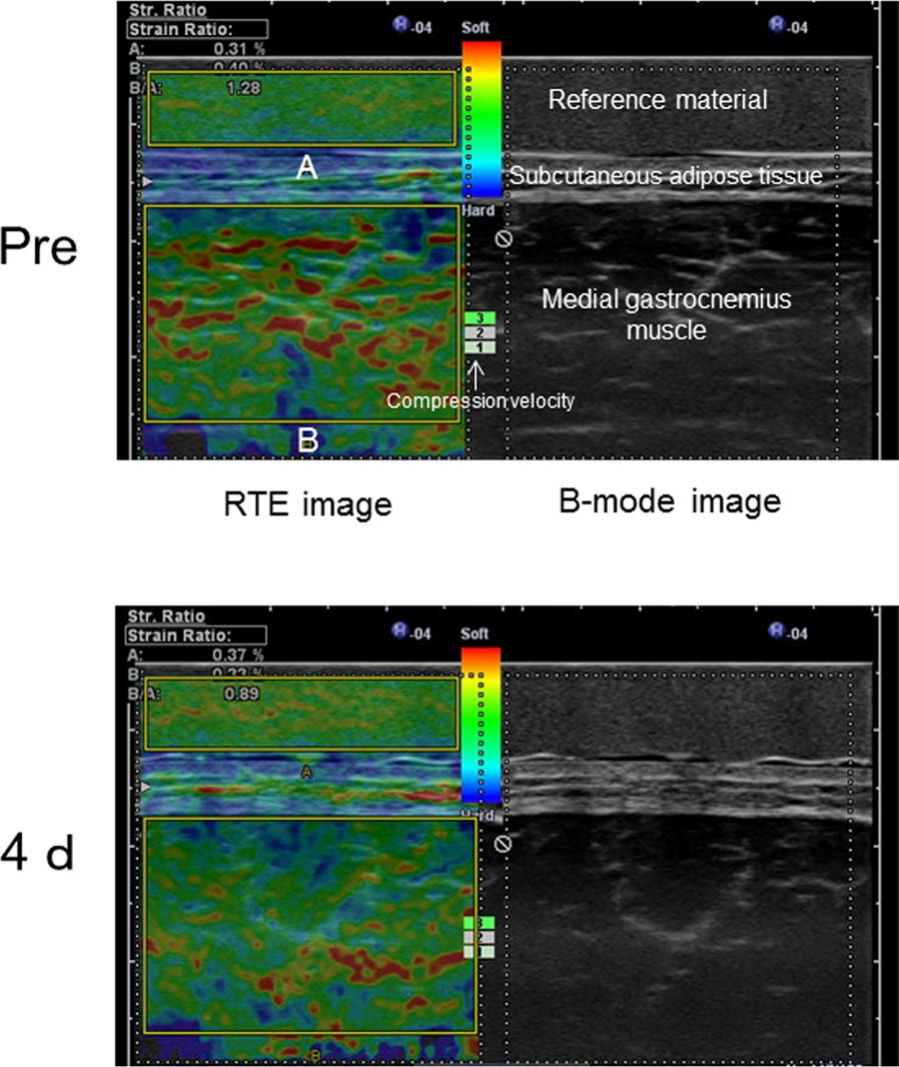
Changes in the axial RTE image superimposed on the B-mode image of a representative subject before and at day 4 after exercise. The rectangular ROIs were placed within the MG (*B*) and a reference material (*A*) on an RTE image obtained at each measurement time. The *color code* indicated the relative strain of the tissues and the reference material to the compression force within the field of view, and ranged from *red* (soft) to *blue* (hard), with *green* indicating average strain. The compression velocity during scan was expressed as a numeric scale ranging from levels 1–6 on the lateral part of the RTE image (recommended velocity by manufacturer: 3–5 levels). Compared with the pre-exercise RTE image, the post-exercise RTE image showed a decrease in the *red* region and an increase in the *blue* and *green* regions in the ROI in the MG.

**Figure 3:**
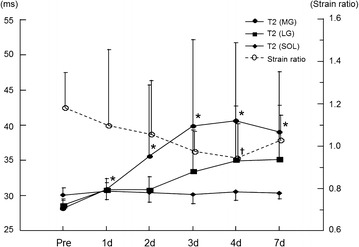
Time course of changes in T2 values in the triceps surae muscles (MG: medial gastrocnemius; LG: lateral gastrocnemius; and SOL: soleus) and strain ratio of the MG before and after exercise. Values are represented as mean ± SD. *P < 0.01: significantly greater T2 values compared with pre-exercise value in the MG. ^✝^P < 0.01: significantly lower strain ratio compared with pre-exercise value in the MG.

**Figure 4:**
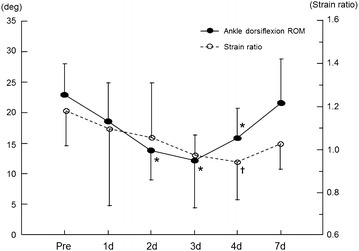
Time course of changes in ankle dorsiflexion ROM and strain ratio of the MG before and after exercise. Values are represented as mean ± SD. *P < 0.01: significantly decreased ankle dorsiflexion ROM compared with pre-exercise value. ^✝^P < 0.01: significantly lower strain ratio compared with pre-exercise value in the MG.

**Figure 5:**
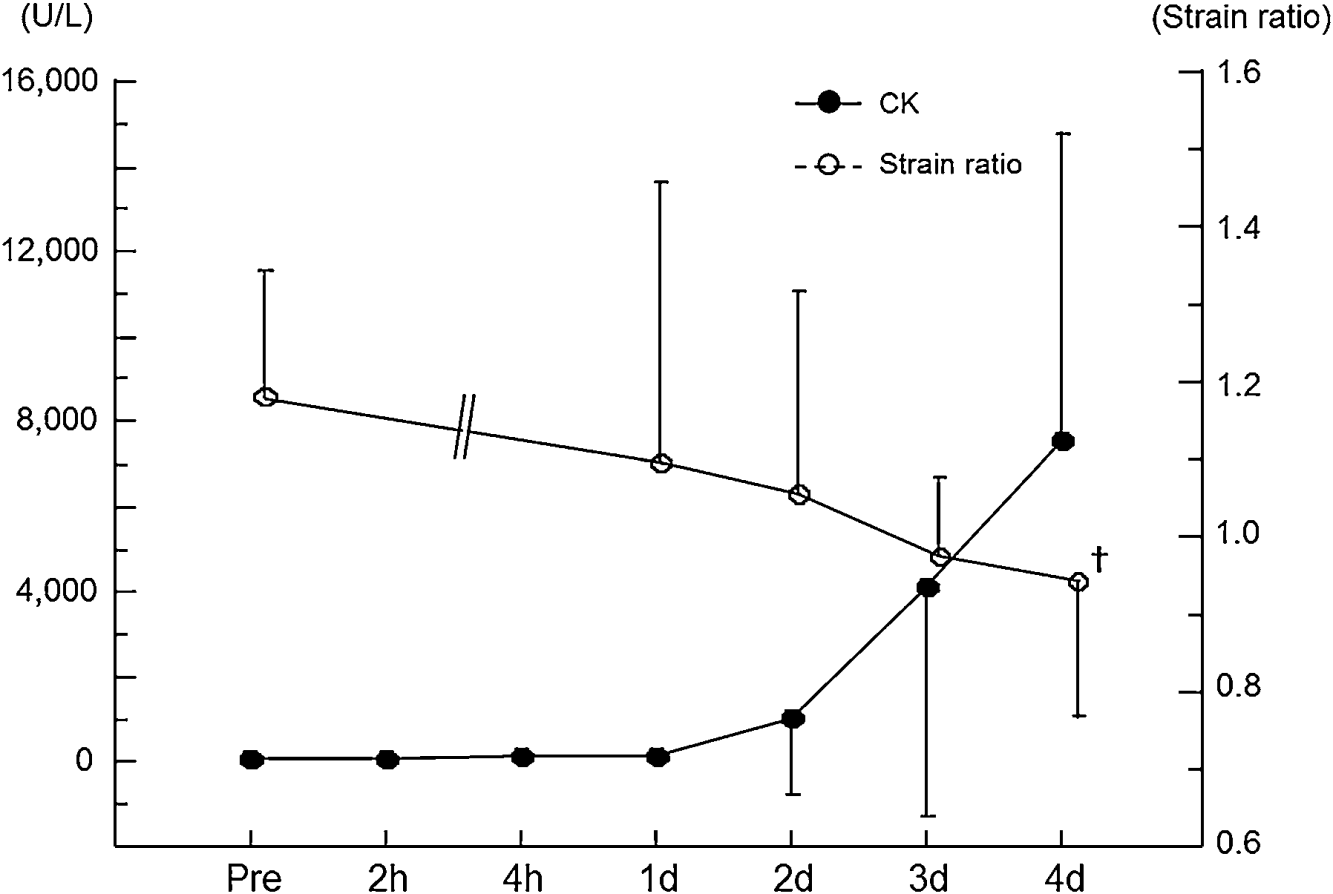
Time course of changes in serum CK activity and strain ratio of the MG before and after exercise. Values are represented as mean ± SD. ^✝^P < 0.01: significantly lower strain ratio compared with pre-exercise value in the MG.

**Figure 6:**
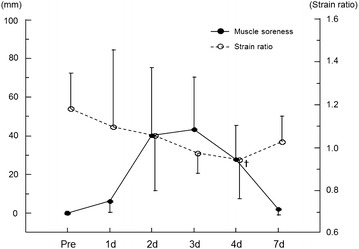
Changes in muscle soreness level and strain ratio of the MG before and after exercise. Values are represented as mean ± SD. ^✝^P < 0.01: significantly lower strain ratio compared with pre-exercise value in the MG.

Figure 2 shows the changes in muscle hardness in a representative subject as color-coded RTE images; increased hardness of the MG was visually apparent on day 4 post-exercise. The strain ratio of the MG showed a significant time effect over the entire measurement period (Friedman test, P < 0.05). This value was significantly lower than the pre-exercise value (1.19 ± 0.17) on day 4 after exercise (0.92 ± 0.17) (Wilcoxon test, P < 0.01, Figures 3, 4, 5, 6). On the other hand, the strain ratio of the MG showed no significant correlation with the T2 value of the MG (rs = 0.54, P = 0.189), ankle dorsiflexion ROM (rs = −0.21, P = 0.6), or muscle soreness level (rs = 0.18, P = 0.661) on day 4 post-exercise.

### MR Imaging

**Figure 7:**
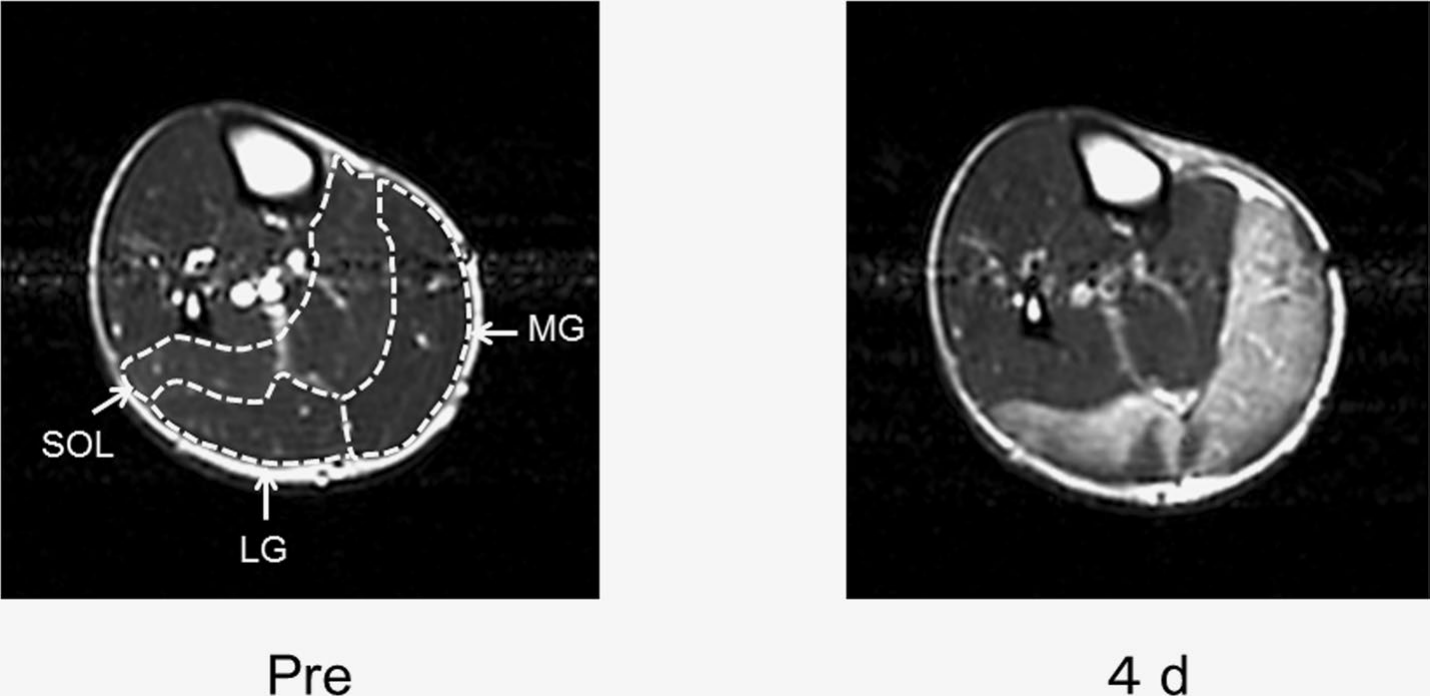
Axial T2-weighted images (repetition time = 2,500 ms, echo time = 80 ms) of the right leg before and at day 4 post-exercise. Increased signal intensity was more prominent in the MG on post-exercise T2-weighted image (MG: medial gastrocnemius; LG: lateral gastrocnemius; and SOL: soleus). This increased signal intensity reflects increased fluid content within the MG. In particular, the size of the MG apparently increased at day 4 after exercise, likely due to the edema formation.

There was a significant main effect of time on the T2 value of the MG (Friedman test, P < 0.05), but no significant time effect on the T2 values of the LG and SOL was apparent. The T2 values of the MG were significantly higher than the pre-exercise value (28.2 ± 1.3 ms) on days 1 (30.9 ± 1.5 ms), 2 (35.6 ± 10.1 ms), 3 (39.9 ± 12.3 ms), 4 (40.7 ± 11.0 ms), and 7 (39.1 ± 8.5 ms) after exercise (Wilcoxon test, P < 0.01, Figure 3). Figure 7 shows axial T2-weighted images of the right leg before and 4 days after exercise in the same subject shown in Figure 2. Region of increased signal intensity in the MG was more pronounced on T2-weighted image obtained on day 4 post-exercise.

### Ankle dorsiflexion ROM

We found a significant main effect of time on the ankle dorsiflexion ROM (Friedman test, P < 0.05). The ankle dorsiflexion ROM was significantly lower than the pre-exercise value (22.9° ± 5.1°) on days 2 (13.8° ± 4.8°), 3 (12.1° ± 7.7°), and 4 (15.8° ± 4.9°) after exercise (Wilcoxon test, P < 0.01, Figure 4).

### Serum CK activity

There was a significant main effect of time on the serum CK value (Friedman test, P < 0.05). However, the Wilcoxon test did not reveal any significant increase from the pre-exercise value at any post-exercise time point (Figure 5).

### Muscle soreness

None of the subjects had any muscle soreness before exercise. There was a significant main effect of time on the muscle soreness level (Friedman test, P < 0.05). However, there was no significant difference in the degree of muscle soreness among the post-exercise time points (Figure 6).

## Discussion

Eccentric muscle contraction frequently causes structural damage to the exercised muscles (Friden et al. 1983; Jones et al. 1986; Beaton et al. 2002). In our study, this exercise-induced muscle damage was indicated indirectly by increased serum CK activity, muscle soreness, decreased dorsiflexion ROM, and elevated T2 value in the MG. In addition, this damage might contribute to the elevated muscle hardness of the MG in RTE measurement. On the other hand, no significant correlations were found between the muscle hardness evaluated by RTE and the other damage-related variables.

Our results showed a significant effect of time on serum CK activity, suggesting that the repeated eccentric muscle contraction damaged muscle fibers. The blood CK level serves as an indirect index of eccentric contraction-induced muscle damage (Jones et al. 1986; Nosaka and Clarkson 1996; Nosaka et al. 2001; Beaton et al. 2002; Yanagisawa et al. 2003b, 2011a; Paulsen et al. 2005; Guilhem et al. 2013; Koch et al. 2014; Fulford et al. 2015). Muscle fibers are subject to greater mechanical stress during eccentric muscle contraction. CK efflux should result from the loss of sarcolemmal integrity (increased permeability and/or breakdown of the membrane surrounding the muscle cell) due to the mechanical stress imposed by the repeated eccentric muscle contraction (Friden et al. 1983; Nosaka and Clarkson 1996; Koch et al. 2014).

The MG showed significantly elevated T2 values on days 1–7 after exercise, but the LG and SOL had no significant change throughout this study. This non-uniformity among the triceps surae muscles is likely attributable to variation in the degree of muscle fiber disruption caused by repeated eccentric muscle contraction. The MG is most heavily recruited by dynamic ankle plantar flexion exercise with the knee extended among the plantar flexors (Yanagisawa et al. 2003a). The T2 elevation in the MG mainly reflects increased intramuscular fluid due to muscle damage. The serum CK level tended to increase in a delayed manner, being higher 3 and 4 days post-exercise. We can infer that elevated interstitial osmotic pressure due to the efflux of CK into the extracellular space might have drawn blood plasma into the interstitial spaces of the damaged muscles, resulting in formation of edema in the MG (Friden et al. 1983; Nosaka and Clarkson 1996; Koch et al. 2014). Moreover, eccentric contraction-induced muscle damage attracts inflammatory cells such as neutrophils and macrophages to the site of damage to remove disrupted muscle tissue fragments and initiate muscle repair (Beaton et al. 2002; Paulsen et al. 2005; Peake et al. 2005; Kanda et al. 2013). In general, neutrophils infiltrate damaged muscle within several hours after eccentric exercise, and are replaced by blood monocyte-derive macrophages within approximately 24 h post-exercise (Peake et al. 2005). The infiltration of inflammatory cells from the blood vessel is accompanied by fluid effusion (Peake et al. 2005). In addition, various cytokines that act as inflammatory mediators that increase vascular permeability are produced in the damaged muscle within several hours after repeated eccentric contraction (Paulsen et al. 2005; Peake et al. 2005). Therefore, elevation of the extravascular osmotic pressure and/or increased vascular permeability as a result of this inflammatory process may be also associated with edema formation within the MG, especially early after exercise. Indeed, T2 elevation would be associated with not only increased intramuscular water content, but also inflammatory responses and direct muscle fiber damage (Yanagisawa et al. 2003b, 2011a; Guilhem et al. 2013; Lacourpaille et al. 2014; Fulford et al. 2015).

The present study also showed significantly decreased ankle dorsiflexion ROM on days 2–4 after exercise. Previous studies have reported decreased joint ROM for several days after eccentric exercise as an indirect evidence of muscle damage (Nosaka and Clarkson 1996; Murayama et al. 2000; Nosaka et al. 2001; Ingham et al. 2010). The decrease in ROM observed in the present study is expected to reflect increased longitudinal muscle stiffness in the ankle plantar flexors, partly as a result of a damage-induced increase in the intracellular Ca^2+^ level in muscle fibers after eccentric exercise (Whitehead et al. 2001; Hoang et al. 2007; Reisman et al. 2009). Judging from post-exercise T2 elevation, the damaged MG might contribute greatly to the decreased ankle dorsiflexion ROM after exercise.

The strain ratio of the MG was significantly lower on day 4 post-exercise, indicating increased muscle hardness. Similarly, Murayama et al. (2000), using the tissue pressure method, found that muscle hardness measured in the relaxed-joint position increased on days 4 and 5 after eccentric exercise and that this change seemed to be associated with local edema resulting from muscle damage. We hypothesized that there would be a pathophysiological relationship between increased hardness and local edema within the exercised muscle. However, the present study found no significant correlation between the strain ratio and the T2 value in the MG on day 4 post-exercise. Furthermore, the strain ratio of the MG showed no significant decrease on day 1, 2, 3, or 7 after exercise regardless of the significantly increased T2 values at these time points. Therefore, this study suggests that local edema does not contribute to the elevation of muscle hardness. Lacourpaille et al. (2014) also showed that muscle shear elastic modulus increased after eccentric exercise, but the increase had no clear relationship with T2 elevation (fluid accumulation) in the damaged muscle. Moreover, although elevated muscle hardness has been suggested to reflect an increase in muscle stiffness after eccentric exercise (Murayama et al. 2000), we found no clear correlation between decreased strain ratio and reduced joint ROM on day 4 post-exercise. Murayama et al. (2000) revealed that the hardness of the elbow flexor muscles was greater in the elbow-extended position than in the elbow-flexed position. Lacourpaille et al. (2014) also reported that the increase in muscle shear elastic modulus after eccentric exercise was more pronounced and persisted longer at long than at short muscle length. However, we did not measure the hardness of the MG in a forcibly stretched state. This may explain why the effect of increased longitudinal muscle stiffness was not largely reflected on the hardness of the MG in our study.

The time course of changes in muscle hardness may be affected by either relaxation or stretch of the muscle during the measurement. Niitsu et al. (2011) showed with RTE that the hardness of the biceps brachii muscle measured in the elbow-extended position peaked on day 2 after eccentric elbow flexion exercise and then decreased until day 4 post-exercise. Murayama et al. (2000) also reported, using the tissue pressure method, that the hardness of elbow flexor muscles peaked earlier after eccentric elbow flexion exercise when measured in the elbow-extended position (day 3 post-exercise) than when measured in the elbow-relaxed position (day 5 post-exercise). These studies showed earlier post-exercise elevation in muscle hardness than observed in our study. Unlike our study, the aforementioned studies measured muscle hardness in positions in which the muscle fibers were stretched. As longitudinal muscle stiffness frequently peaks within 2 days after eccentric exercise (Howell et al. 1993; Hoang et al. 2007; Yanagisawa et al. 2011a), increased longitudinal muscle stiffness may contribute significantly to muscle hardness early post-exercise.

The present study showed a significant main effect of time on the muscle soreness level, but no significant difference in the degree of muscle soreness among the post-exercise time points. Subjects reported muscle soreness during mechanical stimulation, such as muscle extension during walking, but almost no soreness during relaxation. Muscle soreness likely reflects ultrastructural muscle fiber damage, and mechanoreceptor stimulation may contribute to the perception of soreness (Weerakkody et al. 2001). We found no significant correlation between increased muscle hardness and muscle soreness on day 4 after exercise. Andersen et al. (2006) also reported increased hardness and decreased pressure pain threshold in an eccentrically exercised muscle, but could not detect a clear correlation between the two variables. Therefore, elevated muscle hardness may not be a critical determinant of muscle soreness.

The present study did have some limitations. First, our small sample size might have limited our statistical power. Second, as mentioned above, muscle stiffness along the longitudinal axis of the muscle is expected to contribute strongly to muscle hardness (Murayama et al. 2000). Therefore, the strain ratio of the MG in our study might have decreased more dramatically if we had measured the muscle hardness with the muscle in a forcibly stretched state. Third, although we evaluated the muscle hardness and damage only in the muscle belly of the MG, the degree of change in these parameters may differ from site to site within the muscle. Actually, Andersen et al. (2006) showed using a tissue pressure method that the hardness of the tibialis anterior greatly increased in the proximal and distal sites of the muscle than in the muscle belly after eccentric exercise with the tibialis anterior. Additionally, Kinugasa et al. (2006) showed using T2 values that the medial, anterior and distal portions of the MG were more activated by a calf-raise exercise. Their findings suggested heterogeneous distribution of activity within the MG during the calf-raise exercise. Shin et al. (2009) also revealed that the mechanical stress imposed by eccentric muscle contraction was heterogeneously distributed within the healthy human MG. Taken together, multi-positional estimation of muscle hardness and damage might be a better way to delineate the condition of the muscle after eccentric exercise and to determine the relationship between these parameters. Finally, although the reliability and validity of the quantitative method for measuring muscle hardness using RTE has been verified (Yanagisawa et al. 2011b; Chino et al. 2012), the findings of RTE measurement should be compared to those of other imaging techniques, such as MR elastography and supersonic shear imaging.

## Conclusions

RTE revealed that muscle hardness increases after strenuous resistance exercise including repetitive eccentric muscle contraction. However, the increased muscle hardness did not correlate with muscle damage indicators, such as muscle edema, decreased joint ROM and muscle soreness.

## Abbreviations

CK: creatine kinase
LG: lateral gastrocnemius
MG: medial gastrocnemius
MR: magnetic resonance
ROI: region of interest
ROM: range of motion
RTE: real-time tissue elastography
SD: standard deviation
SOL: soleus

## References

[CR1] Andersen H, Arendt-Nielsen L, Danneskiold-Samsoe B, Graven-Nielsen T (2006). Pressure pain sensitivity and hardness along human normal and sensitized muscle. Somatosens Mot Res.

[CR2] Beaton LJ, Tarnopolsky MA, Phillips SM (2002). Contraction-induced muscle damage in humans following calcium channel blocker administration. J Physiol.

[CR3] Chino K, Akagi R, Dohi M, Fukashiro S, Takahashi H (2012). Reliability and validity of quantifying absolute muscle hardness using ultrasound elastography. PLoS One.

[CR4] Crenshaw AG, Gerdle B, Heiden M, Karlsson S, Friden J (2000). Intramuscular pressure and electromyographic responses of the vastus lateralis muscle during repeated maximal isokinetic knee extensions. Acta Physiol Scand.

[CR5] Drakonaki EE, Allen GM (2010). Magnetic resonance imaging, ultrasound and real-time ultrasound elastography of the thigh muscles in congenital muscle dystrophy. Skeletal Radiol.

[CR6] Friden J, Sjostrom M, Ekblom B (1983). Myofibrillar damage following intense eccentric exercise in man. Int J Sports Med.

[CR7] Friden J, Sfakianos PN, Hargens AR (1986). Muscle soreness and intramuscular fluid pressure: comparison between eccentric and concentric load. J Appl Physiol (1985).

[CR8] Friden J, Sfakianos PN, Hargens AR, Akeson WH (1988). Residual muscular swelling after repetitive eccentric contractions. J Orthop Res.

[CR9] Fulford J, Eston RG, Rowlands AV, Davies RC (2015). Assessment of magnetic resonance techniques to measure muscle damage 24 h after eccentric exercise. Scand J Med Sci Sports.

[CR10] Guilhem G, Hug F, Couturier A, Regnault S, Bournat L, Filliard JR (2013). Effects of air-pulsed cryotherapy on neuromuscular recovery subsequent to exercise-induced muscle damage. Am J Sports Med.

[CR11] Hoang PD, Herbert RD, Gandevia SC (2007). Effects of eccentric exercise on passive mechanical properties of human gastrocnemius in vivo. Med Sci Sports Exerc.

[CR12] Howell JN, Chleboun G, Conatser R (1993). Muscle stiffness, strength loss, swelling and soreness following exercise-induced injury in humans. J Physiol.

[CR13] Ingham SA, van Someren KA, Howatson G (2010). Effect of a concentric warm-up exercise on eccentrically induced soreness and loss of function of the elbow flexor muscles. J Sports Sci.

[CR14] Jones DA, Newham DJ, Round JM, Tolfree SE (1986). Experimental human muscle damage: morphological changes in relation to other indices of damage. J Physiol.

[CR15] Kanda K, Sugama K, Hayashida H, Sakuma J, Kawakami Y, Miura S (2013). Eccentric exercise-induced delayed-onset muscle soreness and changes in markers of muscle damage and inflammation. Exerc Immunol Rev.

[CR16] Kinugasa R, Kawakami Y, Fukunaga T (2006). Quantitative assessment of skeletal muscle activation using muscle functional MRI. Magn Reson Imaging.

[CR17] Koch AJ, Pereira R, Machado M (2014). The creatine kinase response to resistance exercise. J Musculoskelet Neuronal Interact.

[CR18] Lacourpaille L, Nordez A, Hug F, Couturier A, Dibie C, Guilhem G (2014). Time-course effect of exercise-induced muscle damage on localized muscle mechanical properties assessed using elastography. Acta Physiol.

[CR19] Murayama M, Nosaka K, Yoneda T, Minamitani K (2000). Changes in hardness of the human elbow flexor muscles after eccentric exercise. Eur J Appl Physiol.

[CR20] Niitsu M, Michizaki A, Endo A, Takei H, Yanagisawa O (2011). Muscle hardness measurement by using ultrasound elastography: a feasibility study. Acta Radiol.

[CR21] Nosaka K, Clarkson PM (1996). Variability in serum creatine kinase response after eccentric exercise of the elbow flexors. Int J Sports Med.

[CR22] Nosaka K, Sakamoto K, Newton M, Sacco P (2001). How long does the protective effect on eccentric exercise-induced muscle damage last?. Med Sci Sports Exerc.

[CR23] Paulsen G, Benestad HB, Strom-Gundersen I, Morkrid L, Lappegard KT, Raastad T (2005). Delayed leukocytosis and cytokine response to high-force eccentric exercise. Med Sci Sports Exerc.

[CR24] Peake J, Nosaka K, Suzuki K (2005). Characterization of inflammatory responses to eccentric exercise in humans. Exerc Immunol Rev.

[CR25] Reisman S, Allen TJ, Proske U (2009). Changes in passive tension after stretch of unexercised and eccentrically exercised human plantarflexor muscles. Exp Brain Res.

[CR26] Schuermans J, Van Tiggelen D, Danneels L, Witvrouw E (2014). Biceps femoris and semitendinosus–teammates or competitors? New insights into hamstring injury mechanisms in male football players: a muscle functional MRI study. Br J Sports Med.

[CR27] Shin DD, Hodgson JA, Edgerton VR, Sinha S (2009). In vivo intramuscular fascicle-aponeuroses dynamics of the human medial gastrocnemius during plantarflexion and dorsiflexion of the foot. J Appl Physiol (1985).

[CR28] Steinberg BD (2005). Evaluation of limb compartments with increased interstitial pressure. An improved noninvasive method for determining quantitative hardness. J Biomech.

[CR29] Steinberg B, Riel R, Armitage M, Berrey H (2011). Quantitative muscle hardness as a noninvasive means for detecting patients at risk of compartment syndromes. Physiol Meas.

[CR30] Weerakkody NS, Whitehead NP, Canny BJ, Gregory JE, Proske U (2001). Large-fiber mechanoreceptors contribute to muscle soreness after eccentric exercise. J Pain.

[CR31] Whitehead NP, Weerakkody NS, Gregory JE, Morgan DL, Proske U (2001). Changes in passive tension of muscle in humans and animals after eccentric exercise. J Physiol.

[CR32] Yanagisawa O, Niitsu M, Yoshioka H, Goto K, Itai Y (2003). MRI determination of muscle recruitment variations in dynamic ankle plantar flexion exercise. Am J Phys Med Rehabil.

[CR33] Yanagisawa O, Niitsu M, Yoshioka H, Goto K, Kudo H, Itai Y (2003). The use of magnetic resonance imaging to evaluate the effects of cooling on skeletal muscle after strenuous exercise. Eur J Appl Physiol.

[CR34] Yanagisawa O, Kurihara T, Kobayashi N, Fukubayashi T (2011). Strenuous resistance exercise effects on magnetic resonance diffusion parameters and muscle-tendon function in human skeletal muscle. J Magn Reson Imaging.

[CR35] Yanagisawa O, Niitsu M, Kurihara T, Fukubayashi T (2011). Evaluation of human muscle hardness after dynamic exercise with ultrasound real-time tissue elastography: a feasibility study. Clin Radiol.

